# Additive Effects of Repetition and Predictability during Comprehension: Evidence from Event-Related Potentials

**DOI:** 10.1371/journal.pone.0099199

**Published:** 2014-06-06

**Authors:** Wing-Yee Chow, Sol Lago, Shannon Barrios, Dan Parker, Giovanna Morini, Ellen Lau

**Affiliations:** 1 Department of Linguistics, University of Maryland, College Park, Maryland, United States of America; 2 Basque Center on Cognition, Brain and Language, Donostia – San Sebastián, Gipuzkoa, Spain; 3 Department of Linguistics, University of Utah, Salt Lake City, Utah, United States of America; 4 Department of Hearing and Speech Sciences, University of Maryland, College Park, Maryland, United States of America; The University of Nottingham, United Kingdom

## Abstract

Previous research has shown that neural responses to words during sentence comprehension are sensitive to both lexical repetition and a word’s predictability in context. While previous research has often contrasted the effects of these variables (e.g. by looking at cases in which word repetition violates sentence-level constraints), little is known about how they work in tandem. In the current study we examine how recent exposure to a word and its predictability in context combine to impact lexical semantic processing. We devise a novel paradigm that combines reading comprehension with a recognition memory task, allowing for an orthogonal manipulation of a word’s predictability and its repetition status. Using event-related brain potentials (ERPs), we show that word repetition and predictability have qualitatively similar and additive effects on the N400 amplitude. We propose that prior exposure to a word and predictability impact lexical semantic processing in an additive and independent fashion.

## Introduction

Research in the last three decades has established that the brain actively deploys lexical and contextual information to facilitate word processing during language comprehension. For example, previous work has shown that neural responses to a word are decreased when the word is presented a second time or preceded by a related item in a word list [1]–[4]. This phenomenon is known as lexical priming and has been attributed to eased access to a word in long-term semantic memory after its level of activation has been boosted by the first presentation of that word or a related word. Lexical priming has been observed across different tasks, and it occurs even when the prime is masked from consciousness [5] or when it is embedded in a sentence or discourse [6]–[8]. As such, the mechanism underlying lexical priming is thought to be bottom-up, highly automatized and reflective of the organization of long-term semantic memory [9]–[14].

On the other hand, neural responses to words within a sentence context are also strongly modulated by the fit between these words and their context, such that words that are more predictable in context are processed more easily. The effect of predictability, as in the case of lexical priming, also results in facilitated access to words in long-term semantic memory (e.g., [15]). However, unlike lexical priming, this facilitation is thought to result from the semantic interpretation of the preceding sentence or discourse. Previous research has demonstrated that comprehenders incrementally compute a semantic interpretation of the sentence context and integrate it with their world knowledge to anticipate and pre-activate likely upcoming words on the fly (e.g., [16]–[18]). Further, previous research has also shown that the effect of prediction is not reducible to lexical semantic priming (e.g., [19]–[20]). As such, sentence context is thought to facilitate lexical semantic processing through a top-down mechanism that, in contrast with lexical priming, relies on the combinatorial computation of contextual information and is sensitive to task demands and subject to strategic control.

However, less is known about how the mechanisms that compute lexical and sentence-level information work in tandem to facilitate lexical semantic processing in comprehension. Studying their combined effect is important because discourses in natural language are structured around semantically coherent structures or topics ([21]–[23]), and there are systematic topic-to-word and word-to-word relationships. For example, if a text or passage has “finances” as its topic, the probability of occurrence of a word like “bank” will be high, and the appearance of this word might in turn make it more likely that words like “federal” and “reserve” will also appear in the discourse. Therefore, examining how lexical and contextual information combine to facilitate the processing of words in sentences should lead to a better understanding of how these sources of information are used in everyday language situations, where both are expected to jointly aid in the comprehension of discourse. In the current study, we address this issue by examining the interaction of word repetition and word predictability. In particular, we ask: how does recent exposure to a word interact with contextual information during comprehension?

We consider three possible ways in which previous exposure to a word and contextual information can work together during reading comprehension. One possibility is that word processing in sentences is primarily modulated by the information provided by the sentence context, such that repetition only exerts an influence when contextual information is limited and does not strongly predict a word. For example, it has been previously observed that word-level variables such as concreteness and lexical frequency have a weaker impact on neural responses when words are embedded in meaningful sentence contexts (see [24] for discussion). Relatedly, semantic priming effects are larger for words that appear in word lists or in less constraining contexts than in very constraining sentence contexts [25] (see [26] for review).

Alternatively, repetition might affect word processing by strengthening predictions that are already licensed by contextual information. Although we are not aware of a proposal of this nature in the domain of word recognition, analogous proposals have been made in the domains of speech perception and word learning. For example, in speech perception it has been shown that acoustic information is used more effectively if listeners are given contextual information about the type of stimuli they are tested on [27]–[28]. Similarly, infants’ success in using phonetic information to discriminate novel words (e.g. “bin” vs. “din”) in a word recognition task greatly improves when the words’ referential status is provided (e.g., by being paired with an object), as compared with when the words are presented in isolation [29]–[30]. Both these examples suggest that low-level perceptual information might be more useful when deployed together with rich contextual information. Analogously, repetition and sentence predictability may facilitate lexical semantic activation in a supra-additive fashion, with the effect of repetition being larger for words that are more predictable in a given sentence context.

We contrast these alternatives with the possibility that word repetition and context predictability have independent and additive effects on lexical semantic activation. According to this view, recent exposure to a word always results in facilitated processing of that word in a sentence context, regardless of its predictability. This view predicts that word repetition and context predictability should have an additive influence on lexical semantic activation even if the word previously appeared outside of the sentence context. One class of computational models that could predict such a pattern are cache-based natural language models, in which information about which words have appeared recently in the discourse is maintained in a running cache and combined with information about the current sentence context to yield an estimate of word probability [31]. A recent example of independent contributions of lexical and sentential context in comprehension comes from work suggesting that the frequencies of the multiple entries of category-ambiguous words are computed independently of the context they appear in [32].

We examine these alternatives using the N400 component as a measure of lexical semantic activation. The N400 is a broad negative deflection of the event-related potential (ERP) that starts 200–300 ms after a word has been presented and peaks after approximately 400 ms [33]. Although a precise interpretation of the processes indexed by this component is still under debate (see [34] for review) we adopt here the proposal that the N400 reflects activation of the semantic features of the long-term memory representations that are associated with a lexical item [15], [35]. According to this view, the N400 response to a word indexes how easy or hard it is to retrieve this word from long-term semantic memory. Correspondingly, words that have been encountered recently or that are more expected are associated with a reduction in the N400 amplitude, with localization evidence suggesting that this differential activity is generated in regions of temporal cortex involved in representing lexical and conceptual information [34], [36]. Therefore, the N400 provides a good implicit measure for examining the mechanism by which lexical repetition and contextual information combine to impact word activation during comprehension.

### Previous Work

Many studies have reported N400 reductions due to word repetition (e.g., [7], [8], [37]) and predictability (e.g., [38]–[40]). A few studies have examined the joint influence of these factors, and they have mainly concluded that repetition effects are context-dependent. One group of studies has focused on cases where repetition violates sentence-level constraints, in examining how repetition of proper names is modulated by co-referential constraints [41]–[43]. These studies have shown that a repeated name like “Daniel” in the sentence “*At the office Daniel moved the cabinet because Daniel* …” elicits a larger N400 than in the control sentence “*At the office Daniel and Amanda moved the cabinet because Daniel* …”. The larger N400 for the repeated name in first sentence has been attributed to a violation of coreference constraints, under which when a referent is prominent in the discourse (as in the first sentence) it should be referred to using a pronoun instead of a proper name. The N400 effect elicited by repeated names that infelicitously refer to a prominent antecedent has been called the *repeated name penalty*. This finding has been taken to argue that repetition effects are context-dependent, and that they can be overriden by higher level processes.

Similarly, a previous study using a memory paradigm found that when both a target word and its context are repeated, word repetition reduces the N400 to incongruous words only, consistent with repetition effects being dependent on context [7]. In this study, participants read sentences in which the final word was either highly predictable in a congruous sentence or highly unpredictable in an incongruous sentence. Predictability was assessed offline using the cloze procedure, in which a separate group of participants were asked to provide completions to sentence fragments [44]; in this study the sentence-final words had a cloze probability greater than 0.75 in the predictable conditions and a cloze probability of 0 in the unpredictable conditions. Participants were asked to memorize the sentence-final words (first presentation) and were then given the sentence frames and asked to recall the missing final words (recall test). Afterwards, they read the same set of sentences for a second time (second presentation). Comparing ERPs across the first and second presentations, the authors found that repetition led to a reduced N400 response for incongruous words but not for congruous ones, yielding a statistical interaction between predictability and repetition. They suggested that lexical repetition does not affect normal sentence processing, but it can facilitate the processing of incongruous words.

However, one potential concern about these previous studies is that they manipulated word repetition in such a way that the second occurrence of the word was infelicitous, either because it violated a pragmatic constraint [41]–[43], or because the entire sentence context was repeated in a rather artificial way [7]. In contrast, word repetition in typical language comprehension presumably takes place in congruous sentences, and while repetition of words is fairly common, repetition of whole sentences is not. More importantly, these studies manipulated lexical repetition and contextual variables in a non-orthogonal manner, such that the effects of the manipulated variables could not be clearly dissociated. Specifically, in the repeated name penalty studies [41]–[43], repetition rendered the sentences infelicitous, and therefore the observed N400 patterns may have reflected both the effect of repetition and of the incongruity of the target words in context. In the study by Besson and colleagues [7], since the target words appeared in identical sentences across both presentations, the repetition of the contexts likely changed the predictability of the target words on second presentation. Thus, unpredictable words might have become much more predictable when repeated in the same context. For example, even though the word ‘socks’ is unexpected in a sentence like ‘*I like my coffee with cream and…*’, it is likely to be more expected when the same sentence appears for the second time. Therefore, it is unclear whether the observed N400 reduction was due to lexical repetition of the target, to its increased predictability in the sentence context, or both.

In order to dissociate between the effects of word and sentence repetition, in a later study Besson and Kutas examined the effects of repetition of low cloze probability words (cloze probability <28%) and varied whether they appeared in the same or in different sentence contexts across presentations [37]. They showed that when the sentence context was changed, word repetition did not modulate the N400. As a result, they concluded that word repetition led to a reduced N400 response only when contexts are also repeated. However, since the predictability of the target words was not manipulated in the study, these results cannot address our question about the combined effects of word repetition and predictability on lexical semantic activation. Furthermore, the finding that lexical repetition leads to an N400 reduction only when the word appears in identical contexts across presentations seems at odds with previous findings in the single-word literature, where words repeated in word lists are consistently associated with reduced N400 responses [4], [45]–[50]. Although target words are preceded by different words across the first and second presentations, repetition consistently leads to an N400 reduction, suggesting that repetition can facilitate lexical semantic activation even when it occurs across different contexts.

In summary, although previous work has established that lexical repetition and contextual information can impact lexical semantic activation during comprehension, questions about how they work in tandem remain unanswered as there are discrepancies across studies and paradigms. Below we present a new paradigm that addresses some of these concerns in order to examine the interaction between lexical and contextual information during sentence processing.

### The Present Study

The current study investigated whether recent exposure to a word interacts with contextual information during comprehension by examining the joint effect of word repetition and predictability on the N400 amplitude. We devised a novel paradigm in which word repetition always occurred in non-repeated contexts. Since word repetition and predictability can be manipulated orthogonally in this paradigm, we can avoid some of the ambiguities in interpretation encountered by previous studies.

**Figure 1:**
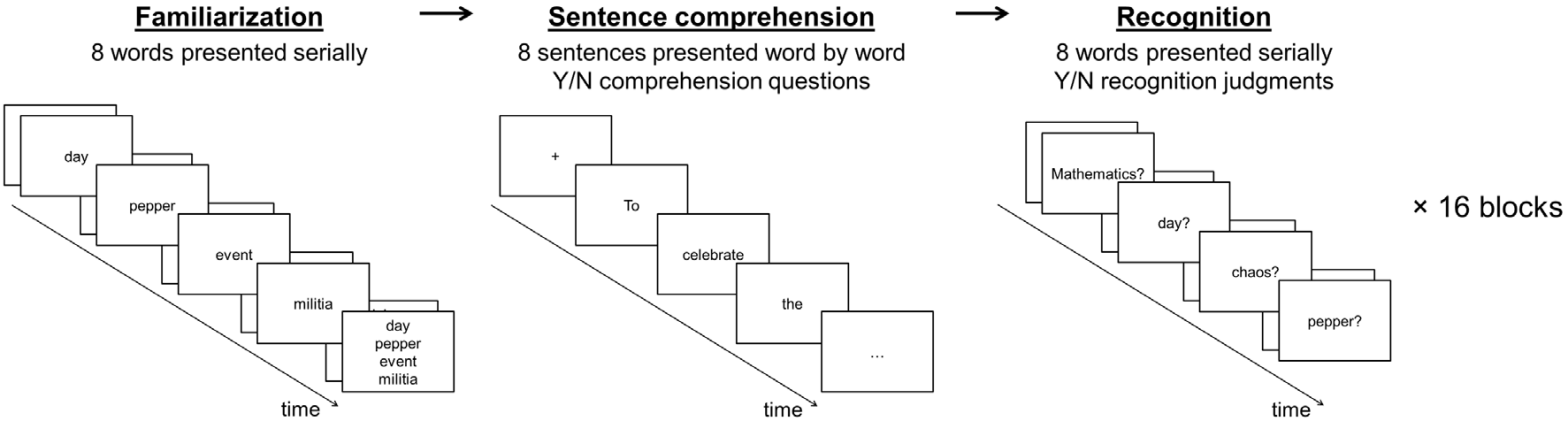
The three phases used in the experimental paradigm: familiarization (left), sentence comprehension (center) and memory recognition test (right).

As illustrated in Figure 1, the paradigm consisted of a familiarization phase, a reading comprehension phase and a recognition test phase. In the familiarization phase participants were asked to study a set of words for a later recognition memory test. Since differences at initial memory encoding have been shown to affect the amplitude of the N400 during later recognition [51]–[52], we presented the words in isolation rather than in sentence contexts to minimize systematic encoding differences. In between the familiarization and the recognition memory test phase, participants read a list of sentences for comprehension while their electroencephalogram (EEG) was recorded. We manipulated whether the target words had been studied in the familiarization phase or not (old vs. new) as well as their predictability in the sentence context (expected vs. unexpected), which was operationalized as their cloze probability. In order to avoid floor effects on the N400 amplitude (cfr. [26]), we used expected target words of intermediate cloze probability (7.9–39.5%). Representative sample items are shown in (1) to (3) with the target word underlined with expected targets presented to the left of unexpected targets:

1. Vivian wanted to leave the party because she couldn’t stand the noise/drinks and the rowdy crowd.
2. Brian looked all over the house for his missing keys/watch before leaving for work.
3. The doctor realized that the patient would need a transplant/miracle in order to survive.

We divided the experimental session into 16 short blocks, each containing 8 words for familiarization, 8 sentences for comprehension, and 8 words for the recognition memory test. This was motivated by two main considerations: we reasoned that while a long delay might substantially weaken the effects of repetition, a delay that is too short (i.e., one with too few trials per block) might make it apparent to the participants that half of the studied words would reappear in a sentence within the same block. This might have encouraged participants to predict that the studied words would reappear during sentence comprehension, rendering our experimental manipulations non-orthogonal, as repeated words would also have been more predictable. For these reasons, we piloted the experiment with different block sizes. We decided to pursue 8-trial blocks since they were short enough for participants to perform the memory recognition task well above chance-level without making apparent whether and/or when a studied word would reappear in the sentences within the same block. Further, since multiple-trial blocks afford variable time lags between the first and second presentations of a word, they allow us to better model the fact that word repetition may occur over any number of sentences in written texts or speech in real-life settings.

Different patterns of results are predicted by each of the three hypotheses discussed in the Introduction about how lexical repetition and contextual information combine to impact lexical semantic activation. If recent exposure to a word and predictability affect lexical semantic activation independently, they should have an additive effect on the N400 amplitude. Alternatively, if lexical repetition facilitates lexical semantic activation only when a word is not predictable in a given context, then the effect of repetition on the N400 should be larger for unexpected words than expected words. This would be consistent with observations in previous studies on semantic priming (e.g., [25]). Lastly, if word repetition can strengthen or reinforce the predictions that arise from the sentence context, then the effect of repetition on the N400 should be larger for expected words than unexpected words.

## Methods

### Participants

Twenty-four students (12 female, mean age = 21.1 years, range = 18–28 years) from the University of Maryland, College Park participated in the current study. Informed consent was obtained in all cases. Participants were right-handed, native English speakers with normal or corrected-to-normal vision.

### Ethics Statement

This study was conducted with the approval of the University of Maryland, College Park Institutional Review Board (UMCP IRB). All participants gave written consent and were paid 20 USD for their participation in accordance with the policies of UMCP IRB.

### Materials

Stimuli for reading comprehension consisted of 128 sentence item sets. We orthogonally manipulated the repetition status (old vs. new) and the predictability (expected vs. unexpected) of the target word in the sentences. A target word was considered ‘old’ if it was presented in the preceding familiarization phase and ‘new’ otherwise. Cloze probabilities for the experimental sentences were obtained in a norming study. Participants were 114 student volunteers at the University of Maryland, College Park. They were asked to provide the best continuations for 220 sentence frames. A total of 180 sentence fragments for which the maximum cloze probability was below 40% were selected to form the sentences for the EEG study. Expected target words had an average of 22.5% cloze probability (range = 7.9–39.5%); unexpected plausible target words were selected from words that were provided exactly once (0.9% cloze probability) in the norming study. Thus, all experimental sentences were semantically congruous. The sentences were extended beyond the target word in order to avoid wrap-up effects.

The stimuli for the familiarization phase consisted of 128 words. Half of these words were presented as target words in sentences for reading comprehension and the other half were words selected to match the lexical frequency of the target words (average log frequency of targets: 3.20; fillers: 3.16; [53]). For the recognition task the stimuli also consisted of 128 words, half of which were presented in the familiarization phase, while the other half were new words matched in average frequency. Thus, only half of the 64 studied words appeared as targets in sentences for reading comprehension.

The sentence items and the words for the familiarization and recognition tasks were distributed in four presentation lists using a Latin square design. Each list contained 128 words for familiarization and recognition respectively, along with 128 sentences (32 per condition), each paired with a corresponding Yes/No comprehension question. The overall ratio of Yes/No target response was 1∶1 in each presentation list. Each list was presented to six participants. The materials were presented in 16 blocks, each containing 8 words for familiarization, 8 sentences for reading comprehension and 8 words for recognition. The order of blocks and the materials within each phase in each block were pseudorandomized across participants.

### Procedure

As illustrated in Figure 1, participants were instructed to memorize the words presented during the familiarization phase for a later recognition task. In between each familiarization and recognition phase they were asked to read sentences attentively and to answer comprehension questions about those sentences.

In the familiarization phase words were presented in two sequences of four, and each sequence was followed by a screen on which all four words were presented together. In each sequence words were presented individually at the center of the screen for 600 ms, followed by 400 ms of blank screen. At the end of each sequence the four words reappeared on the screen together. Participants were told to press a button to proceed to the second sequence (or to the next phase if they had seen both sequences) when they had memorized the words. In the sentence comprehension phase, sentences were presented one word at a time at the center of the screen. Each sentence was preceded by a fixation cross that appeared for 500 ms. Each word appeared on the screen for 300 ms, followed by 230 ms of blank screen. The last word of each sentence was marked with a period, followed by a comprehension question 1000 ms later. Participants were instructed to avoid eye blinks and movements during the presentation of the sentences and to answer the comprehension questions by pressing one of two buttons. In the recognition phase, each trial consisted of a word presented at the center of the screen. Participants indicated whether the word had been presented in the familiarization phase of that block by pressing one of two buttons. The next trial began automatically after they had responded.

Prior to the experimental session, participants completed a practice block with 8 words for familiarization, 4 sentences for reading comprehension and 8 words for recognition. The experimental session was divided into 16 blocks, with short pauses in between. Including set-up time, an experimental session lasted between 1.5 and 2 hours.

### EEG Recording

EEG was recorded continuously from 29 AgCl electrodes mounted in an electrode cap (Electrocap International): midline: Fz, FCz, Cz, CPz, Pz, Oz; lateral: FP1, F3/4, F7/8, FC3/4, FT7/8, C3/4, T7/8, CP3/4, TP7/8, P4/5, P7/8, and O1/2. Recordings were referenced online to the left mastoid and re-referenced offline to the average of the left and right mastoids. The electro-oculogram (EOG) was recorded at four electrode sites; vertical EOG was recorded from electrodes placed above and below the left eye and the horizontal EOG was recorded from electrodes situated at the outer canthus of each eye. Electrode impedances were kept below 5 kΩ. The EEG and EOG recordings were amplified and digitized online at 1 kHz with a bandpass filter of 0.15–100 Hz.

### ERP Data Analysis

All trials were evaluated individually for EOG or other artifacts. Trials contaminated by artifacts were excluded from the averaging procedure. This affected 9.5% of experimental trials. A digital 40 Hz low-pass filter was used on all data to reduce high-frequency noise. Event-related potentials were computed separately for each participant and each condition for the 1000 ms window after the onset of the target word relative to a 100 ms pre-stimulus baseline.

Analyses focused on 18 electrodes that could be evenly distributed across the topographic factors of interest: F3, FZ, F4, FC3, FCZ, FC4, C3, CZ, C4, CP3, CPZ, CP4, P3, PZ, P4, O1, OZ, and O2. Statistical analyses on average voltage amplitudes were conducted in R [54] separately for two time windows selected based on existing literature on the N400 component and visual inspection: 300–400 ms for the N400, and 600–800 ms for later differences. We conducted Type II SS omnibus repeated measures ANOVAs that fully crossed repetition (old vs. new) and predictability (expected vs. unexpected) with anteriority (anterior vs. central vs. posterior) and laterality (left vs. midline vs. right). Electrodes were distributed across the topographic factors as follows: left-anterior: F3, FC3; midline-anterior: FZ, FCZ; right-anterior: F4, FC4; left-central: C3, CP3; midline-central: CZ, CPZ; right-central: C4, CP4; left-posterior: P3, O1; midline-posterior: PZ, OZ; right-posterior: P4, O2. Univariate *F*-tests with more than one degree of freedom in the numerator were adjusted by means of the Greenhouse-Geisser correction.

Further, in order to examine the potential interaction (or the lack thereof) of the effects of repetition and predictability on the N400, we compared two linear mixed-effects models [55], one with and one without an interaction term between predictability and repetition. We asked whether the model with an interaction term provided a better fit for the N400 data as compared to a model without it. Using the *lme4* package [56] we fitted two linear mixed-effects models to the 300–400 ms time-window averages in the midline-posterior region. Both models had by-subject and by-item random intercepts, but the simpler model only had repetition and predictability as fixed effects while the more complex model included an additional repetition-by-predictability interaction term. We then conducted a likelihood ratio test (e.g., [57]) to determine if the more complex model provided a better fit to the data.

### Behavioral Data Analysis

Participants’ performance on the sentence comprehension and memory recognition tasks was measured. Comprehension accuracy was analyzed using mixed logit models [58] with repetition, predictability and their interaction as fixed effects and by-item and by-subject random intercepts. D-prime (d’) scores [59] were computed to examine participants’ performance on the recognition task. The log-linear correction method described in [60] was used to avoid the appearance of non-finite values in the case of extreme false alarm or hit rates. We conducted a one-sample Wilcoxon test to examine if their average d’ score was above chance.

## Results

### Behavioral Results

Participants performed well on both the sentence comprehension questions and the recognition memory task. They answered the comprehension questions with a mean accuracy of 90.8% (old-expected: 91%; old-unexpected: 90.2%; new-expected: 92.6%; new-unexpected: 89.3%). Mixed logit models revealed that comprehension accuracy was higher for sentences in the expected conditions than in the unexpected conditions (expected = 91.8%; unexpected = 89.8%; β = −0.15, *p*(Wald) <.05) but it was not impacted by the repetition status of the target word. The average d’ score on the memory test was 1.83 (SD = 0.82), which was significantly above chance (*p*<0.001). Overall, the behavioral data show that participants performed adequately on both the comprehension and the memory components of the current task.

### Event-related Potentials (ERPs) during Reading Comprehension

**Figure 2:**
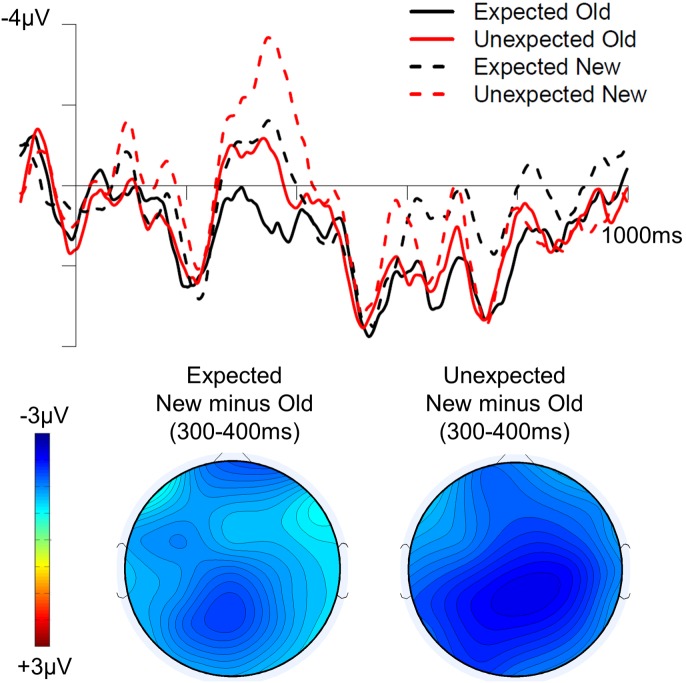
Average ERP waveforms in all four conditions at PZ (top) and topographic maps (bottom) showing the effect of repetition in the expected (left) and unexpected (right) conditions in the 300–400 ms time window.

**Figure 3:**
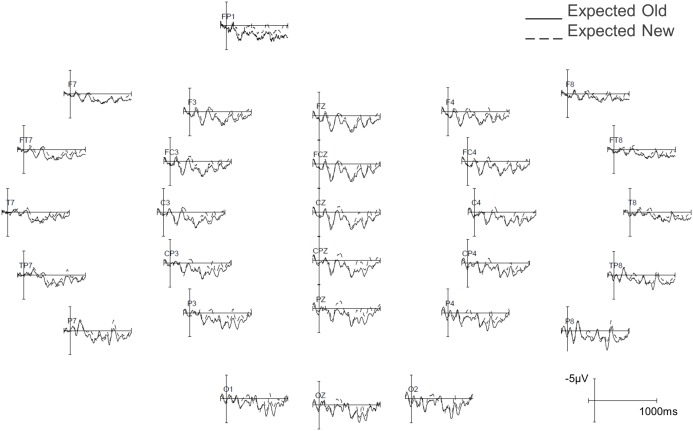
Average ERP waveforms at all 29 scalp sites in the old (solid line) and new (dashed line) expected conditions.

**Figure 4:**
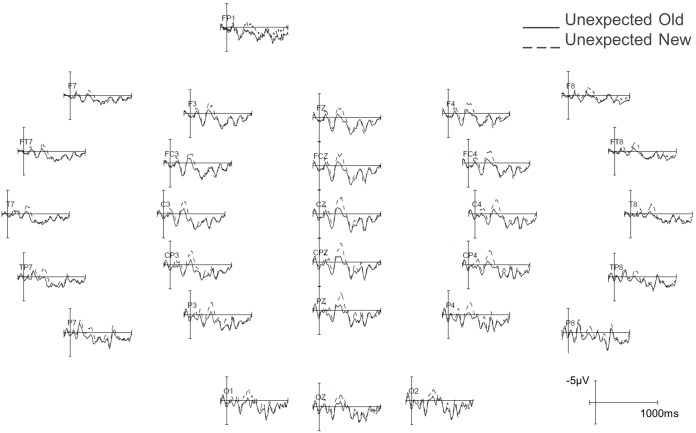
Average ERP waveforms at all 29 scalp sites in the old (solid line) and new (dashed line) unexpected conditions.

Figure 2 shows the grand average ERPs at PZ to target words in all four conditions and the topographic distribution of the repetition effect (new minus old) separately for the unexpected and expected conditions in the 300–400 ms time interval. Figures 3 and 4 show the grand average ERPs across all scalp sites in the expected conditions (old vs. new) and unexpected (old vs. new) conditions respectively. Visual inspection indicates that both experimental factors had a clear effect on the N400: the amplitude of the N400 was reduced for old relative to new target words, and it was also reduced for expected relative to unexpected target words. Table 1 shows the results of the statistical analyses in both time-windows. We report statistics for significant main effects and interactions below.

**Table 1:** Omnibus repeated measures ANOVA F-values at the target word during reading comprehension.

	*df*	300–400 ms	600–800 ms
*Omnibus ANOVA*			
repeat	1,23	14.94**	3.35∧
expect	1,23	11.51**	<1
repeat × expect	1,23	<1	1.76
repeat × ant	2,46	1.07	3.2∧
expect × ant	2,46	1.23	<1
repeat × lat	2,46	<1	<1
expect × lat	2,46	2.31	1.47
repeat × expect × ant	2,46	<1	<1
repeat × expect × lat	2,46	<1	<1
repeat × ant × lat	4,92	2.26∧	<1
expect × ant × lat	4,92	1.37	<1
repeat × expect × ant × lat	4,92	1.96	<1

*repeat* = repetition; *expect* = expectancy; *ant* = anteriority; *lat* = laterality.

**p<.01 *p<.05 ∧.05<*p*<.1.

**Figure 5:**
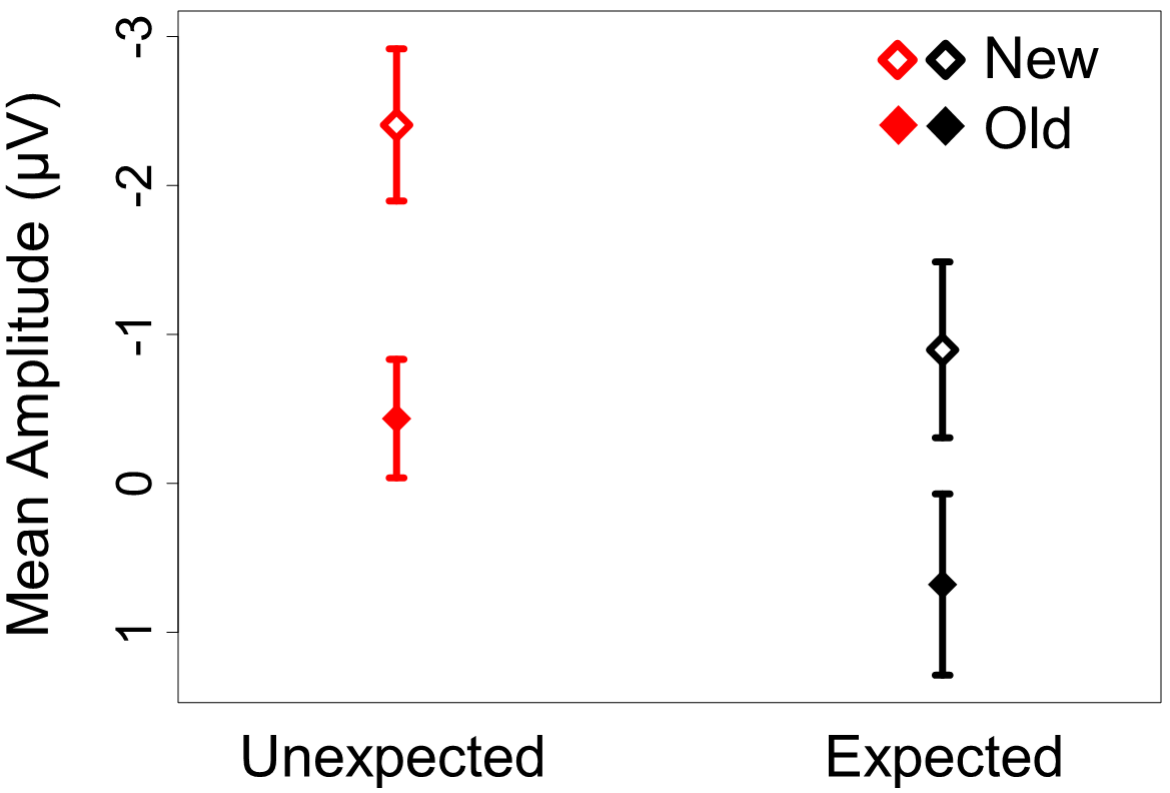
Interaction plot showing additive effects of repetition and predictability in mean amplitude during the N400 time window (300–400 ms) at the midline posterior region. The expected and unexpected conditions are plotted in black and red respectively.

Consistent with these observations, omnibus repeated measures ANOVA in the 300–400 ms interval revealed significant main effects of both repetition and predictability. The main effect of repetition was driven by ERPs being less negative in the old than in the new conditions (*F*(1,23)  = 14.94, *p*<.01). The main effect of predictability was driven by ERPs being less negative in the expected than in the unexpected conditions (*F*(1,23)  = 11.51, *p*<.01). Both effects were broadly distributed across the scalp, as shown by the lack of significant interactions with topographic factors. Crucially, no significant interaction between these two factors was obtained (all *F*s<2). This additive pattern is displayed in Figure 5, which shows the average ERP amplitude in the 300–400 ms interval in the midline posterior region. The main effect of predictability and repetition and the absence of an interaction between them also held in two alternative time-windows that have been previously used to assess N400 effects (200–400 ms and 300–500 ms).

In order to further examine the additivity of the effects of predictability and repetition, two linear mixed-effects models were fitted to the N400 data in the midline posterior region. Both models had predictability and repetition as fixed effects and by-subject and by-item random intercepts; one of them included an additional interaction term between predictability and repetition. A likelihood ratio test revealed that the more complex model did not provide a significantly better fit of the data than the simpler model (*X^2^*_(1)_  = .21; *p* = .64), and thus the removal of the interaction term from the model was statistically justified. In fact, the near-zero *X^2^* value shows that including an interaction term in the model hardly improves its fit of the data at all. Even though both analyses used a null hypothesis significance testing approach and thus neither allowed us to confirm the null hypothesis, the results from both analyses are consistent in showing that repetition and predictability modulated the size of the N400 in an additive fashion.

In the 600–800 ms interval, omnibus repeated measures ANOVA revealed a marginally significant main effect of repetition (*F*(1,23)  = 3.35, *p* = .08) and a marginal repetition × anteriority interaction (*F*(2,46)  = 3.2, *p* = .07). These effects were not followed up further as they failed to reach statistical significance and they were not predicted by any of the hypotheses examined in the current study.

## Discussion

The aim of the present study was to investigate how recent exposure to a word interacts with the processing of contextual information during sentence comprehension. We devised a paradigm that allowed us to orthogonally manipulate a word’s repetition status and its predictability in a sentence context. In line with previous studies that have examined the effects of lexical repetition and predictability separately [7]–[8], [37]–[38], [40], [61], both factors led to a reduction in the N400 amplitude in the present study. In addition, we show evidence that their effects are additive, such that the N400 response to a recently encountered word is reduced by a similar amount regardless of the word’s predictability in context.

Our observation that word repetition reduces the N400 response to both expected and unexpected words differs from the results of Besson and colleagues [7], who found that repetition reduced the N400 response to unexpected words only. We attribute this discrepancy to two primary differences in the experimental paradigm and materials used across studies. First, while all of the expected words in Besson et al.’s study had high cloze probability, in the current study only expected words of intermediate cloze probability were used. Since highly predictable words elicit small N400 responses, they might result in floor effects that could have led to the absence of a repetition effect in the expected condition in Besson et al.’s study [7] (cfr. [26]). Further, unlike Besson et al.’s sentence repetition task, in the current paradigm the target words were first studied in a word list, and they were presented in a sentence context only during the reading comprehension phase. This not only minimized potential encoding differences between expected and unexpected words during familiarization, but also eliminated potential concerns about the effects of context repetition. Therefore, the present paradigm permitted a truly orthogonal manipulation of a word’s repetition status and its predictability in context, and we believe that it was these methodological improvements that allowed us to observe repetition effects on the N400 response to expected as well as unexpected words.

The current findings of additive effects of repetition and predictability on N400 amplitude are consistent with two hypotheses. First, memory for recent words and contextual information may impact lexical semantic processing via distinct mechanisms that independently modulate a word’s activation level. Specifically, we propose that the ease of lexical semantic processing is modulated by (i) bottom-up, exposure-driven changes to the word’s residual activation level in long-term semantic memory, and (ii) top-down, pre-activation of the word’s semantic features as a result of the semantic interpretation of the preceding context. Repetition facilitates lexical semantic processing because recent exposure to a word increases its activation level in memory; lexical semantic processing is facilitated for predictable words because comprehenders incrementally compute a semantic interpretation of the sentence context and integrate it with their world knowledge to anticipate and pre-activate likely upcoming words. Under this view, previous exposure to a word and linguistic predictions act on the same representations stored in long-term semantic memory, but they exert their effects through distinct and independent mechanisms.

Alternatively, contextual information and recent exposure to a word may both impact lexical semantic activation via a predictive mechanism. Crucially, the facilitative effect of repetition on lexical semantic activation may not be fully attributable to residual activation of previously encountered words. Instead, lexical semantic activation may be facilitated upon repetition because having recently encountered a word directly strengthens comprehenders’ expectations for that word to appear again; this is essentially the assumption also made by cache-based natural language processing models [31]. Under this view, lexical repetition and predictability modulate lexical semantic activation via a shared neurocognitive mechanism: previous exposure to a word as well as sentential interpretation are incorporated into predictive computations, which in turn facilitate word recognition by pre-activating lexical semantic representations in memory. Some evidence for such a view has been demonstrated in the domain of face recognition [62], where the facilitative effect of repetition is modulated by the likelihood that repetitions occur in the experiment. If this view is correct, then the additivity of these factors (or the lack thereof) provides information about how different types of evidence are combined in generating predictions about upcoming words, rather than indicating that these factors impact lexical semantic activation through different and independent mechanisms.

In sum, it is important to distinguish between a view that holds that sentential context and prior exposure to a word modulate lexical semantic activation via two distinct mechanisms (prediction and residual activation from recent exposure, respectively), and the possibility that both factors modulate lexical semantic activation via the same mechanism (prediction). Future research will be needed to address this question.

Meanwhile, the current findings provide no support for the other two proposals outlined in the Introduction, both of which predicted a significant interaction between lexical repetition and context predictability. First, the current results are inconsistent with the proposal that prior exposure facilitates lexical semantic processing during comprehension primarily by strengthening predictions that are already licensed by the sentence context. This account predicted a larger effect of repetition for expected than unexpected words. However, the present results suggest that word repetition facilitates lexical semantic activation of target words both when their occurrence is predictable by contextual information and when it is not.

The present results also do not support the proposal that lexical semantic activation during comprehension is primarily modulated by the predictions afforded by the sentence context, with lexical factors exerting an influence only when words are not predictable by context. This proposal draws on the close relationship between repetition and semantic priming, and the previous observation that semantic priming has a larger effect on the N400 amplitude in words lists or in less constraining sentence contexts than in more constraining sentence contexts [25]. The current findings, however, suggest that repetition priming displays a different profile than what has been previously reported for semantic priming, as it affects both expected and unexpected words alike.

It is important to note that differences between repetition and semantic priming have previously been noted in behavioral studies. For example, while repetition effects are known to occur over relatively long intervals, semantic priming effects tend to be short-lived [63]. In addition, repetition effects are reliably obtained across different tasks, whereas semantic priming effects are reduced when tasks are changed, for example, from lexical decision to naming [64]–[66]. These differences have led to the proposal that repetition and semantic priming might reflect different underlying processes. For example, it has been suggested that while repetition effects might be due to an automatic lexical activation mechanism, semantic priming might partly be the result of top-down prediction [64], [67]–[68]. However, as the current study did not directly contrast interactions with repetition priming and with semantic priming, future work will be needed to examine the extent to which this discrepancy might be explained by differences in experimental paradigms across studies.

Importantly, one assumption in the present study is that the effect of predictability on the N400 reflects the behavior of a predictive mechanism that makes use of contextual information to pre-activate words with higher cloze probability, facilitating lexical semantic processing. This is in line with the view that comprehenders make probabilistic predictions about likely upcoming words [16], [61] and the finding that the N400 is sensitive to a word’s predictability across the full range of possible cloze probability values [16], [69]–[70]. However, like most previous studies that examined the effects of cloze probability (e.g., [35], [38]–[40]; c.f. [71]), the current study was not designed to determine exactly how the expected target words (or their semantic features) were pre-activated by sentence context. While target words may have been pre-activated based on the message-level information of the sentence context, they may also have been pre-activated partly due to their lexical relationship with other words or phrases in the context. Therefore, in the current study, a word may have been more expected on the basis of its relation to the sentence context as a whole, to words and phrases in the sentence context, or both.

Finally, the methodology used in the current study has several important limitations. First, although rarely discussed in this light, it is possible that repetition also modulates the P3 component, which is sensitive to the detection of improbable stimuli (for review see [72]–[73]), and this effect may not have been distinguishable from the N400 effect if these two components had overlapped in time. In the current study, we cannot rule out the possibility that repetition modulated the P3 in addition to the N400 response to the target words during sentence comprehension, but we consider it unlikely given some crucial differences between our paradigm and those that have been used to elicit P3 effects in the past. First, previous studies have shown that P3 effects are strongly attenuated when a participant’s attention is directed away from the task in which the targets are embedded [74]–[75], and in the current study the studied words were not task-relevant during the sentence comprehension phase. In fact, participants were explicitly instructed to base their recognition decisions on whether the words had been presented in the familiarization phase and to ignore whether the words appeared in the sentences. Furthermore, unlike most studies on the P3 component, which have used tasks that required participants to track one (or two) very infrequent targets embedded in a stream of stimuli, the present study presented eight targets in each block, and each target had a 50% chance of occurring in a sentence during the reading comprehension phase. Therefore, we believe that the N400 effect of repetition in the current study is unlikely to be due to an overlapping P3 effect.

Second, the repetition manipulation in the present paradigm was not naturalistic, in the sense that participants did not encounter the repeated words in passages, but instead studied them before the sentence-reading phase. This was done in order to orthogonally manipulate the effects of repetition and predictability, and also to allow a more direct comparison with previous studies, which also used a memory paradigm [7], [37]. We believe that this paradigm provides a first step towards examining how lexical and contextual variables work in tandem to facilitate the processing of words in sentences; future research will need to develop more naturalistic methods for examining their interaction.

In summary, the current study presents a new experimental paradigm for examining the combined effects of lexical repetition and predictability. We demonstrate that contextual information and memory for recently encountered words have qualitatively similar and additive effects on the N400 response during sentence comprehension. A better understanding of how these variables work in tandem will inform theories about how language is processed in everyday language situations, where sentential context and previous occurrences of a word are expected to jointly aid in the comprehension of discourse. Therefore, by allowing a truly orthogonal manipulation of different factors that are known to impact lexical semantic activation, the current paradigm provides a useful tool for future research on word recognition during language comprehension.
